# Accelerometer Measurements: A Learning Tool to Help Older Adults Understand the Importance of Soft-Landing Techniques in a Community Walking Class

**DOI:** 10.3390/s25154546

**Published:** 2025-07-22

**Authors:** Tatsuo Doi, Ryosuke Haruna, Naoyo Kamioka, Shuzo Bonkohara, Nobuko Hongu

**Affiliations:** 1 Dynamic Sports Medicine Institute, 1-10-28, Nishi-Shinsaibashi, Chuo-ku, Osaka 542-0086, Japan; loco1952@dream.jp (T.D.); haruna@dsmi.jp (R.H.); 2 Department of Nutrition, Graduate School of Human Life and Ecology, Osaka Metropolitan University, 3-3-138, Sugimoto, Sumiyoshi-ku, Osaka 558-8585, Japan; 3 Department of Physical Therapy, Faculty of Health Science, SBC Tokyo Medical University, 5-8-1 Akemi, Urayasu-shi 279-8567, Chiba, Japan; kamioka@sbctmu.ac.jp (N.K.); bonkohara@sbctmu.ac.jp (S.B.)

**Keywords:** older adults, walking acceleration, landing impact, gait assessment, community walking program for elderly

## Abstract

**Highlights:**

**What are the main findings?**

- A positive correlation was found between step length and upward acceleration.
- The practicality of measuring upward acceleration reflects the vertical impact forces (landing impact) among older adults participating in a community walking class.

**What is the implication of the main finding?**

- It is possible to reduce physical strain by shortening your step length.
- The physical load placed on your body can be seen numerically in an upward acceleration results sheet.

**Abstract:**

When people overextend their step length, it leads to an increase in vertical movement and braking force. The overextension elevates landing impacts, which may increase pain in the knees or lower back. The objective of this study was to examine the effects of soft-landing walking techniques in a 90 min, instructor-led group class for older adults. To evaluate a landing impact, an accelerometer measurement system (Descente LTD., Tokyo, Japan) was used to measure a participant 10 meter (m) of walking. Assessment outcomes included the average number of steps, step length, upward acceleration which reflects the landing impact, and survey questions. A total of 223 older adults (31 men, 192 women, mean age 74.4 ± 5.7 years) completed the walking lesson. Following the lesson, participants decreased their step lengths and reduced upward acceleration, along with an increased step count. The number of steps increased, and a positive correlation (*r* = 0.73, *p* < 0.01) was observed between the rate of change in step length and upward acceleration. Over 95% of participants gave high marks for practicality and understanding the accelerometer measurements. The information derived from this study will provide valuable insight into the effectiveness of soft-landing techniques as a promotion of a healthy walking program for older adults.

## 1. Introduction

Regular physical activity is crucial for maintaining quality of life in older adults [1] while also preventing sarcopenia [2], cognitive decline [3], falls, and fractures [4,5,6]. Walking is the most prominent form of physical activity. Thus, extending walking abilities is believed to increase healthy life expectancy. The World Health Organization‘s 2020 guidelines on physical activity and sedentary behavior indicate an inverse dose–response relationship between the volume of aerobic physical activity and the risk of physical functional limitations in older adults [7]. Previous studies reported that walking in older adults was characterized by a shorter step length and slower speed [8,9,10], which were associated with various diseases [11,12] and shorter life expectancy [13]. Ardestani et al. investigated the effects of an individual’s walking speed regulating strategy (i.e., increasing cadence or stride length) on lower extremity joint moments when healthy adults between 40 and 60 years of age increased their walking speed from normal to fast [14]. The study revealed that increasing the stride length to walk faster resulted in a significant increase in lower limb joint moments. Participants who increased their cadence to walk faster had little effect on lower limb joint moments. The authors suggested “tailored gait retraining” for individuals with joint pathology. For example, walking faster through increased cadence, rather than increasing the stride length, could be considered as a walking speed regulating strategy to protect the progression of knee osteoarthritis [14]. Edd et al. also demonstrated that stride length is a valuable gait parameter to modify in combination with foot progression angle and step width modifications to reduce medial knee loading. Their findings indicated that a future study is needed to investigate “personalized gait retraining” due to the high intersubject variability in dose–response for osteoarthritic knees [15].

Osteoarthritis is a chronic degenerative condition that affects joints and surrounding tissues, with few effective treatments available to slow the disease progression. Although it can affect any joint, the knees are the most frequently impacted [16]. In the United States, it was estimated that about 14 million people had symptomatic knee osteoarthritis and that half of them were younger than 45 years old [17]. According to a cohort study by Yoshimura et al., it was estimated that 25.3 million people, accounting for 35.6% of those 40 years old or older living in Japan, suffer from knee osteoarthritis [18]. A recent study by Milner et al. reported the acute effects of changes in walking velocity and step lengths on knee joint loads under nine different combinations of gait velocity (e.g., decreased velocity, preferred velocity, increased velocity) and step length (e.g., decreased step length, preferred step length, increased step length) in the groups of healthy normal weight and obese adults [19]. The study used three-dimensional gait analysis data. The results showed that decreasing step length, especially when combined with preferred gait velocity, significantly reduced knee joint loads in both groups. Authors suggested that decreasing step length without reducing velocity during daily walking activities is a way to reduce the cumulative loads on the articular surface of the knee joint during each step [19].

**Figure 1:**
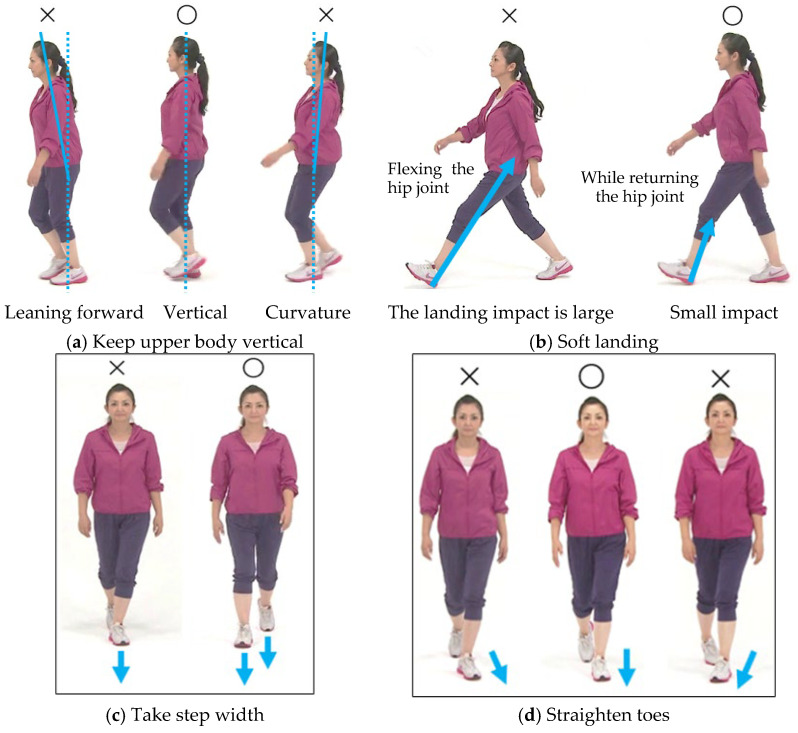
Four tips for the soft-landing techniques [25].

Research on gait changes, such as walking speed and posture in older adults, has been increasingly investigated [20], particularly related to the development of the medial knee osteoarthritis [14,15,19,20,21,22]; however, these studies were mainly conducted within research facilities and not in community program settings. Additionally, there are limited studies focused on walking using soft-landing techniques that could reduce vertical impact forces, which may help lower knee and lower back pain in healthy, older adults [23]. In our previous study, we focused on the fact that many older adults experience knee or lower back pain while they are walking [24]. To help these older adults, we have worked on a walking method/technique to alleviate their pain when they walk. The walking method/technique we implemented for about 15 years in a community walking class, not in a research lab, consisted of the following four tips [25]: (1) keep the upper body vertical to maintain stability in the sagittal plane [26]; (2) extend the lower hip joint before initial contact to achieve a soft landing [27]; (3) take a step width to reduce the knee joint loading on the lower limbs and maintain stability in the frontal plane (i.e., keep your step/foot/leg in line with your hips) [28]; (4) keep the toes pointing straight without twisting knees or ankle joints [15] (Figure 1). The participants in our study revealed the benefits of learning these tips on reduced knee and lower back pains and increased their walking distance [24]. However, we could not teach the participants in community programs about the physical load (i.e., vertical impact forces) on their body. Thus, the purpose of this study was to examine the effectiveness of the learning process using several tools in a 90 min walking class for a group of older adults participating in a community walking program. The program included the following: (1) 10 m gait assessment; (2) watching a video about soft-landing techniques; (3) talking to an instructor; (4) walking practice. We evaluated the following three points: (1) the changes in upward acceleration reflecting the vertical impact forces (landing impact) during their walking; (2) the practicality of the acceleration measurement systems; (3) the understanding of that measurement and the walking tips by the community walking class participants.

## 2. Materials and Methods

### 2.1. Participants

This study was conducted between January 2023 and August 2024. Participants were recruited by sending out a local newsletter about the walking program through a community program office. Participants were included based on the following criteria: (1) adults over 65 years old; (2) no history of a major medical disorder or injury, (3) physical ability (i.e., balance, motor skills, stamina, and strength) to participate in a 90 min walking program; (3) independent walking without any physician-imposed walking restriction due to chronic conditions. Those who did not meet the study’s criteria were excluded. Prior to the study, participants were informed about the purpose of the study, program contents, and methods. All participants verbally gave informed consent to the study instructors. The study was approved by the ethical committee of Osaka City/Metropolitan University (IRB#: 19-52, 21-14) and conducted in accordance with the Declaration of Helsinki.

### 2.2. Walking Program

**Figure 2:**
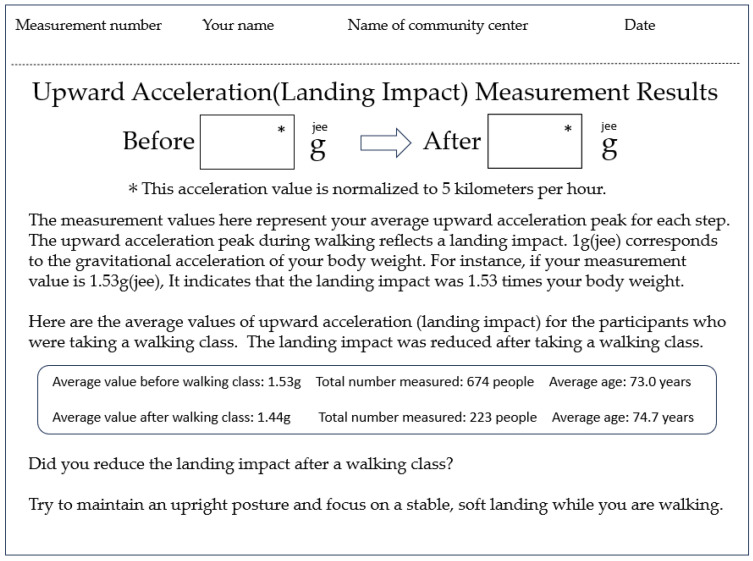
Upward accelerometer measurement result sheet. * 5 km per hour was used based on our previous 10 m walking data. n = 674, means age 73.0 ± 5.6 years, average walking speed = 4.9 ± 0.9 km/h. Normalization acceleration (g) = measured acceleration (g)÷1.3 m/s (walking speed) × 1.39 m/s. Example: 1.5 g (measured upward acceleration) ÷1.3 m/s (walking speed) × 1.39 m/s (fixed speed) = 1.60 g (normalized acceleration). Note: multiplying by a fixed speed (1.39 m/s) may proportionally present any measurement error.

**Figure 3:**
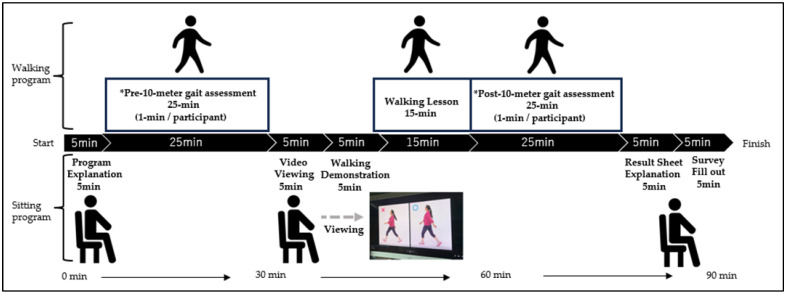
Experimental design of 90 min walking program. * Total duration of 10 m gait assessment is 25 min. It takes 1 min to do the assessment per participant.

The walking program of this study was offered at ten locations in the cities of Amagasaki, Izumi Otsu, Osaka, and Toyota, Japan. Approximately 25 healthy older adults participated at each location. The participants were asked to wear comfortable clothing and shoes to practice the soft-landing walking technique and 10 m walking accelerometer measurement. The procedure was as follows: First, all participants were informed that 10 m accelerometer measurements were to be conducted twice before and after the walking lesson and that they could compare their own results at the end of the walking class. After the explanation and agreement to participate, the participants took 10 m walking accelerometer measurements (before the walking lesson) and received their upward acceleration values (g). Then they viewed a video about walking tips for the soft-landing technique (Figure 1) for about 5 min. After reviewing the video and receiving some questions from the participants, the instructor of the walking program (T.D.) explained and demonstrated four walking tips to the participants. All participants together practiced walking in line at their preferred speed and step length for at least five trials while thinking about these four walking tips (Figure 1). After having a practice walking session, each participant took 10 m walking accelerometer measurements again (after the walking lesson). When all participants finished the measurements, the instructor explained the result sheet (Figure 2) that provided qualitative feedback on landing impact. This whole process may encourage participants to be conscious of landing impact and motive them to actively apply the soft-landing techniques. The participants filled out the survey questions that we have developed based on our experience and knowledge on community walking programs at the end of the walking program (see Appendix A). The total time of the walking program was about 90 min (Figure 3). All participants completed the walking program without any notable fatigue or pain.

### 2.3. 10 m Gait Assessment

**Figure 4:**
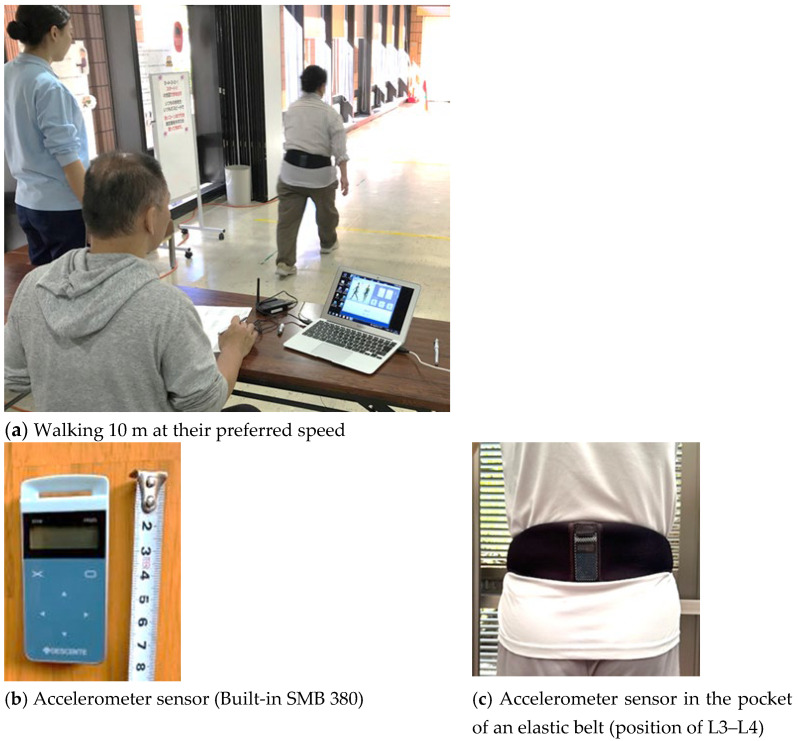
Accelerometer measurement at 10 m walk.

All measurements were conducted by three trained instructors. Participants were instructed to walk for 10 m at their preferred speed before and after taking the walking lesson (Figure 4a). Instructors timed the walking time (seconds) using a stopwatch when the participants crossed the 10 m finish line. Also, the instructors counted the number of steps when the participants reached the finish line and recorded them. Step length (cm) was calculated by the distance that a participant walked (10 m plus the distance that a participant crossed the goal line) divided by the number of steps taken. Then, the step length was recorded as a ratio to height (step length/body height), and normalized data by walking speed (m/s); i.e., step length = (step length (cm)/body height (cm)) ÷ walking speed (m/s). Upward acceleration of each step was measured using the accelerometer sensor (model SMB 380, sensitivity error ±3%) at a sampling frequency of 60 Hz composed of a micro-electro-mechanical system (MEMS) manufactured by Bosch Sensor Tec GmbH (Reut-Lingen, Germany) (Figure 4b). To minimize variability in the sensor placement for each measurement, the accelerometer sensor was inserted into the pocket of an elastic belt worn around the waist, with the belt positioned along the iliac crest so that the sensor adhered closely to the lumbar region (position of L3–L4) (Figure 4c). This approach consistently maintained the positioning of the sensor on the body during a measurement. Furthermore, at the start of each 10 m gait assessment test, while a participant stood still, the Z-axis acceleration value to the gravitational acceleration (1 g) was initialized. This procedure helped to reduce the influence of slight differences in attachment angles. The recorded upward acceleration data was transmitted as a digital signal received by a PC paired via Bluetooth, and the average upward peak acceleration value was automatically calculated (DESCENTE Ltd., Tokyo, Japan).

**Figure 5:**
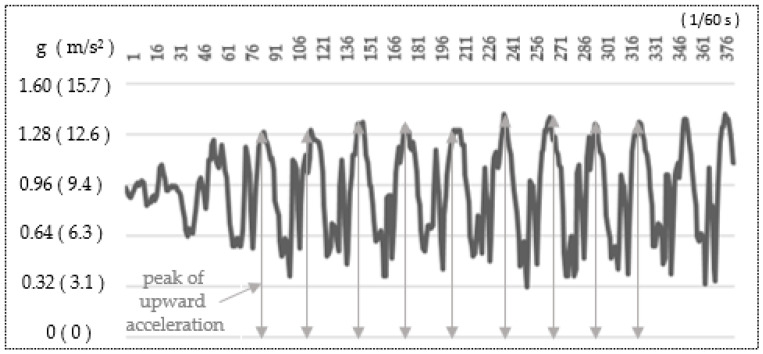
Example of an upward acceleration waveform measured at the lower back during walking. The vertical axis represents upward acceleration in units of g (m/s^2^).

Figure 5 illustrates an example of an upward acceleration measurement waveform. The horizontal axis represents measurement time (unit: 1/60 s), while the vertical axis indicates upward acceleration values. Acceleration is expressed in g (m/s^2^). This conversion follows the International System of Units (SI), where 1 g = 9.81 m/s^2^. The peak detection algorithm was defined as a local maximum peak exceeding 1.2 g in the Z-axis acceleration signal, and a minimum interval of 0.3 s was applied to prevent false detections caused by high-frequency noise. In this study, the peak values of the upward acceleration waveform were analyzed by automatically calculating the average peak value, excluding the first and last two steps.

After the 10 m gait assessment measurement, an upward acceleration result sheet (Figure 2) was provided to each participant. The instructor (T.D.) explained the result sheet to participants as follows: “*Earlier, we had you walk 10 m while wearing this accelerometer on your lower back. The result sheet you received shows the average peak of vertical impact forces detected by the sensor, expressed as a gravitational acceleration value in units of g (jee). When we are standing still, the body is subjected to a gravitational acceleration of 1 g (jee), which equals the force of your own body weight. In other words, the load on your body in a standing posture is calculated as your body weight multiplied by 1 g (jee). However, this gravitational force changes when you are moving. When you walk with greater vertical motion or take longer steps that result in stronger brake forces, the vertical impact forces increases. For example, if the ‘Before’ value is 1.6 g (jee), that means the vertical impact forces corresponds to a load equal to 1.6 times your body weight. If, after taking the walking lesson, the ‘After’ value decreases to 1.5 g (jee), then the load becomes 1.5 times your body weight. This indicates that the vertical impact forces has been reduced by approximately 10% compared to before the lesson, suggesting an improvement in your walking pattern. How was your result?*” By reviewing their own personal result sheet, participants can assess their changes in landing impact before and after the walking instruction. The time required for the 10 m walking accelerometer measurement test and printing of the result sheet was approximately one minute per person. Then, it took approximately 5 min to explain the results sheet to the group. (Note: Strictly speaking, “body weight” refers to mass rather than force. However, in this explanation, we used the term for ease of understanding.)

### 2.4. Questionnaire Measurements

The questionnaire was completed voluntarily and anonymously during the walking lesson, and it included 223 participants from different locations. Demographic characteristics (i.e., age, gender) and body height were self-reported. They were asked questions about their walking style, pain while they were walking, and whether they had a surgical history or not. As part of the lesson, each participant completed a survey at the end of the lesson that asked about program satisfaction. This included whether the lesson could help to change their walking style or not and their understanding of acceleration measurements, and it also provided an opportunity for open-ended responses to both positive and negative aspects, satisfaction with the program, length of program, and suggestions for improvement (see Appendix A: Survey Questions). The purpose of having questionnaire measurements was for capturing participants’ awareness of walking techniques, such as step length, and perceived physical load during walking. Participants were also asked about their experience of accelerometer measurements and receiving the measurement feedback and a result sheet ((Figure 2) upward accelerometer measurement result sheet). The questionnaire items were exploratory tools to supplement our future walking program using accelerometer measurements, and their responses provide insights of participants’ engagement and understanding in the community walking program.

### 2.5. Statistical Analysis

We compared the step length/body height, step count (converted to per minute), the average peak value of upward acceleration (m/s^2^), and walking speed (m/s) before and after having walking lessons. Since the walking speed differed between the pre- and post-instruction measurements, each value (excluding walking speed) was corrected by each participant’s walking speed (m/s). The following formulas were applied to normalize the values: Step length (body height ratio) = (Step length (cm)/Body height (cm)) ÷ Walking speed (m/s); Step count (per minute) = (Step count/Time (s) x60) ÷ Walking speed (m/s); and Upward acceleration= Measured upward acceleration (m/s^2^) ÷ Walking speed (m/s). These normalized values were used for pre- and post-statistical comparisons. Before performing an analysis, the Shapiro–Wilk normality test was performed. The test revealed that all variables except for the post-test walking speed were not normally distributed. (all, *p* < 0.001; except walking speed, *p* = 0.898) Since the data were not normally distributed, for non-parametric analysis, the Wilcoxon signed-rank test was used to compare the step length, the step count, and the average peak value of upward acceleration before and after having the walking lesson. The correlation between the rate of change in step length, the average peak value of upward acceleration, and walking speed before and after the walking lesson was analyzed using Spearman’s rank correlation coefficients. All statistical analyses were performed using SPSS (IBM SPSS Statistics, Version 28, Armonk, NY, USA). The significance level was set at *p* < 0.05. Outcomes showing stronger statistical significance (e.g., *p* < 0.001) were reported to reflect the strength of the evidence.

## 3. Results

### 3.1. Participant Characteristics

A total of 223 participants (31 men and 192 women) with a mean age of 74.7 ± 5.6 years (range 65 to 91 years) attended this study in a community walking class in 10 different locations. Mean body height was 154.4 ± 7.0 cm.

### 3.2. 10 m Gait Assessment Results

Table 1 shows the comparison of 10 m walking measurements between pre- and post-walking lessons. All variables are presented as medians (interquartile range). Step length/body height and upward acceleration were significantly decreased (Step length, *p* < 0.001; Upward acceleration, *p* = 0.005), but step counts/minute significantly increased (*p* < 0.001). No significant changes were observed for the walking speed (m/s) (*p* = 0.899) (Table 1).

**Figure 6:**
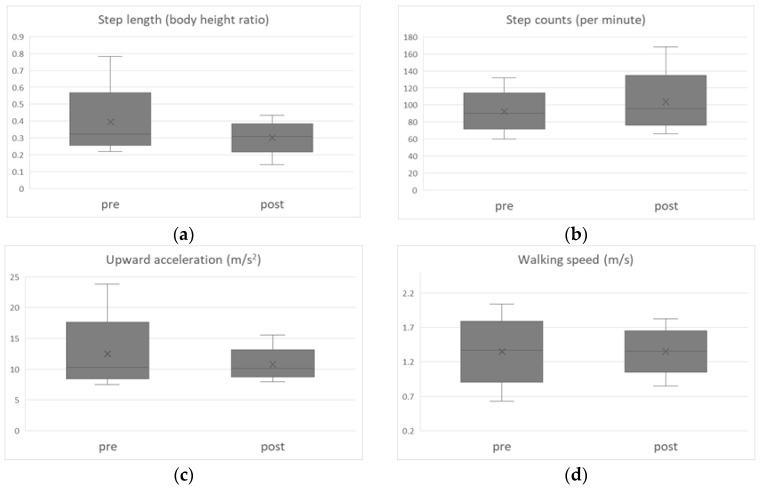
Measurement results for each variable. Boxplots to visualize the pre and post comparisons of each variable. (**a**) Step length (body height ratio), (**b**) step counts (per minute), (**c**) upward acceleration (m/s^2^), (**d**) walking speed (m/s).

Figure 6 shows changes of (a) step length (body height ratio), (b) step counts (per minute), (c) upward acceleration (m/s^2^), and (d) walking speed (m/s) at pre- and post-walking lessons. The findings provide a visual complement to the results of Table 1

**Figure 7:**
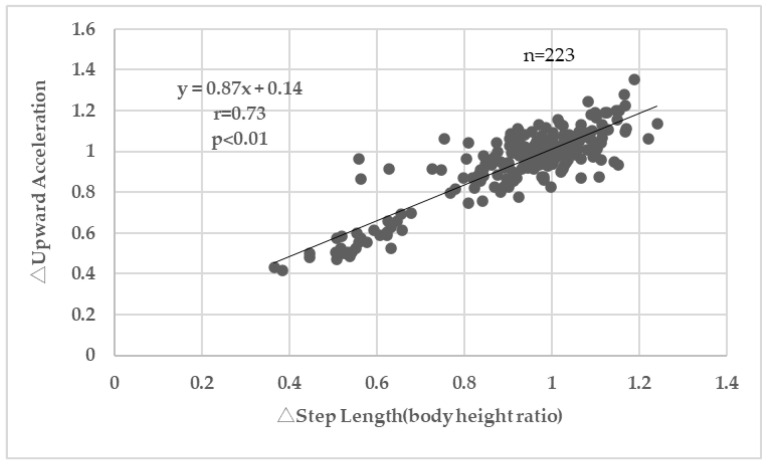
Correlation between the change in step length and upward acceleration. Changes were calculated as post-intervention values divided by pre-intervention values (i.e., post/pre ratios).

Figure 7 shows a positive correlation between the rate of change in step length (normalized by body height) and the rate of change in upward acceleration (r = 0.73, *p* < 0.01). Further examination on the upward acceleration, Table 2 shows step length (body height ratio), step count (per minute), and walking speed (m/s) were all significant predictors of the upward acceleration.

### 3.3. Survey Results

**Figure 8:**
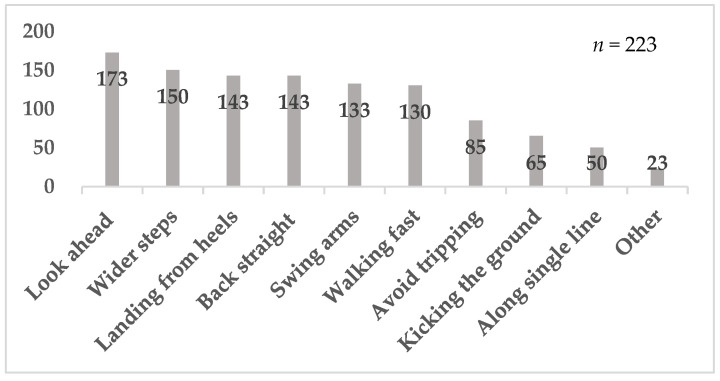
Results of Survey Question #1.

A total of 223 participants answered the survey. Survey Question #1 asked about walking styles that participants focus on while they walk (See Appendix A 1). This question allowed multiple answers. There were 100 or more responses for the following six walking styles that the participants selected: “Looking ahead”, having “Wider steps”, “Landing from heels”, “Back straight”, “Swing arms”, and “Walking fast”. There were 23 “Other” responses. Within the responses, 20 respondents selected “not conscious” while they walked. Three others wrote they are “having a conversation,” “enjoying the scenery,” and “not using unnecessary force” while they walk. (Figure 8)

**Figure 9:**
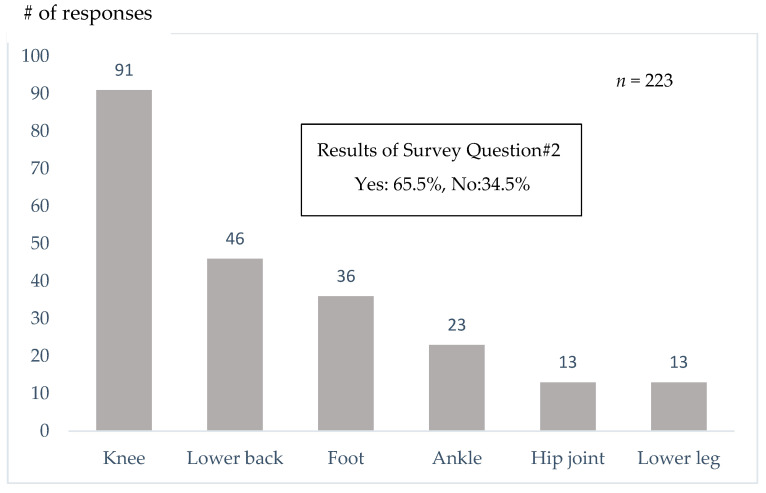
Results of Survey Question #3.

Participants reported being pain-free when they walk, but many of them experience discomfort when they are walking faster or for longer periods. Survey Question #2 asked “Have you ever felt pain while walking?” and 65.5% of the participants in the walking classes reported having pain when they walked. Major sites that participants reported having pain were the knee, lower back and foot (Figure 9).

**Figure 10:**
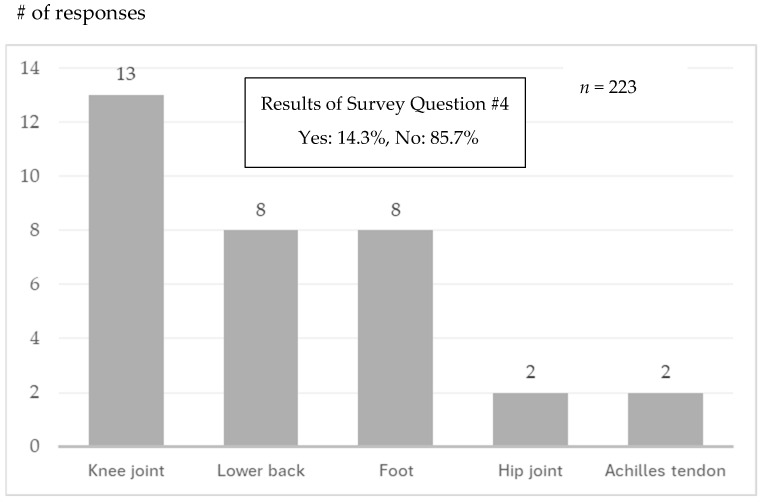
Results of Survey Question #5.

Knee and back pain may worsen and require surgery. Survey Question #4 asked for surgical history. A total of 14.3% of the participants answered that they had a surgical history before taking the walking class. Their surgical sites are shown in Figure 10.

Survey Question# 6 asked whether the participants knew that their way of walking could put a burden on their knees and lower back. A total of 61.9% responded they were unaware of it, but 35.4% answered that their walking does put a burden on their knees and lower back. Next, in Survey Question #7 we asked about the results of their accelerometer measurement values. Almost all participants (96.4%) answered that they understood the measurement values. After reviewing their measurement values, 96.0% of participants responded that they would like to improve their walking styles (Survey Question #8). Regarding the usefulness of the walking program and having 10 m gait assessment measurements, 96.0% answered that an assessment measurement in the walking program was helpful (Survey Question #9). A total of 90.6% expressed a desire to participate in future walking programs in their community (Survey Question #10) (Table 3). There were 22 participants (9.9%) who wrote responses in the open comment section. Of these, 10 comments were about the walking instructions, and another 10 were related to the 10 m gait measurement. All the content was positive, such as “practicing specific walking techniques was helpful” and “walking measurement was beneficial.” Additionally, two participants made requests. One asked for detailed instruction on walking techniques for reducing knee or back pain, while the other requested walking measurement on inclined surfaces.

## 4. Discussion

In Japan, walking instruction programs for older adults are commonly conducted in a group setting at local community centers. This study aimed to develop a practical pro-gram suitable for implementation in such real-world settings. The goal of the walking pro-gram in this study was to enable participants to learn a way of walking that does not place excessive strain on the knees or lower back, and to help them become aware of the physical load associated with walking using several tools. The walking program included (1) video-based instructions, (2) 10 m gait assessment, and (3) hands-on practices to teach soft-landing techniques. In the walking lesson, upward acceleration (Figure 2) was used as an indicator to evaluate the physical load during the participant’s everyday walking. This study focused on the peak of upward acceleration that occurs at the load response phase (Figure 5). This peak is known to be strongly correlated with the vertical component of ground reaction force and can be measured noninvasively using an accelerometer attached to the lower back [29]. Previous studies [30,31] have reported significant correlations between acceleration and ground reaction force, supporting the validity of our measurement approach. In our study all accelerometer measurements were conducted using the same single device with a sensor pocket belt (Figure 4c), and we applied a peak detection algorithm that will help limit measurement errors in pre- and post-walking class comparisons. Our study showed that upward acceleration can be easily obtained in a community setting, making it a highly practical index for general older adult participants.

According to the results in Table 1, both step length and upward acceleration significantly decreased after the walking lesson (*p* < 0.01), while step count significantly increased (*p* < 0.01). Their walking speed did not change (*p* = 0.90). These results suggest that slightly shortening step length and increasing step count reduced vertical impact forces on the lower back. Since no significant change in walking speed was observed, these changes are considered to reflect alterations in gait pattern. In addition, a positive correlation was observed between the rate of change in step length and the rate of change in upward acceleration (Figure 7, *p* < 0.01). This is consistent with previous studies by Ardestani et al. [14], Edd et al. [15], and Milner et al. [19] which reported that shortening step length reduces loading on the lower extremity joints. In the present study, the acquisition of soft-landing techniques through our walking program (90 min) was demonstrated by the acceleration data.

It has been reported that walking with a shorter step length may be related to reduced normal walking functions or an increased risk of falls [32]. It has also been reported that older adults tend to prefer a higher cadence and shorter steps [33]. Therefore, our walking lesson was designed to reduce landing impact while also considering the maintenance of walking function. According to Guy G. Simoneau [28], the range of motion of the hip joint is proportional to walking speed. Based on this, our walking lesson (Figure 1) instructed participants to keep their upper body upright while opening the hips in the sagittal plane during walking. It has also been reported that at initial contact hip extension has already begun, and the vertical heel velocity is controlled to approximately 0.05 m/s just before ground contact. This suggests that the hip movement transitions from flexion to extension at initial contact, which serves to decelerate the heel’s downward motion. Based on these insights, the walking lesson instructed participants to make initial contact with the heel while extending the hip (i.e., while bringing the forward-swing leg back). This slightly shortens the step length but helps to control body drop and leg swing, potentially reducing vertical impact forces (landing impact). In contrast, landing with the hip in flexion increases step length but also increases body drop and swing velocity, which may result in greater impact strength on the knees and lower back.

Yoshimura et al. [18] reported that 35.6% of Japanese people aged 40 and over have knee osteoarthritis, and 54.9% have degenerative lumbar disease. In our questionnaire survey, 65.5% of participants reported having experienced pain while walking, and the most common site of pain was the knee, followed by the lower back (Figure 9). Additionally, 14.3% of respondents had undergone surgery, with the knee joint being the most frequently treated site (Figure 10). Interestingly, 61.9% of participants answered that they were unaware that walking could place stress on the knees or lower back (Table 3). Many participants selected responses such as “wider steps” or “landing from the heels” (Figure 8), suggesting the need for instruction on appropriate walking techniques.

In the present study, an upward acceleration measurement system was used as a tool to assess landing impact within the limited timeframe of the walking class (Figure 3). The measurement was completed smoothly, requiring approximately one minute per participant. In the questionnaire, 96.4% of participants reported understanding the acceleration values, 96.0% found the measurement helpful, 96.0% expressed a desire to improve their walking, and 90.6% were interested in being re-evaluated (Table 3). These findings indicate that upward acceleration measurement is a practical and effective tool for community walking classes. The unit m/s^2^ is unfamiliar to community walking participants and may not be easily understood. Therefore, in our study, values were converted to “g,” and explained with 1 g representing the load equivalent to body weight at rest. We believe this allowed participants to more concretely understand the measured values. This experience highlighted the importance of adapting laboratory-based measurements for practical use in the field to facilitate participant understanding in practice-based research on exercise instruction. Some limitations need to be acknowledged. First, the participants practiced walking and self-evaluated their walking. Although the instructor of the program provided feedback and advice to the group, each participant may need to have feedback from the instructor for better understanding and maintaining their soft-landing techniques. Second, our questionnaire was developed based on the practical experience of the authors, and its psychometric properties (e.g., reliability, validity) were not formally assessed. Therefore, subjective findings from the questionnaire should be interpreted with caution. Third, although we excluded participants with serious cognitive impairments during recruitment, we did not conduct a formal cognitive assessment. Considering the wide age range of participants (65–91 years), the potential heterogeneity in cognitive function may have affected participants’ understanding of instructions or ability to self-evaluate their walking, which could influence the internal validity of our findings. Fourth, in this study women made up majority of participants (86%). This affects the applicability of our findings to the male population. Thus, future studies should actively engage in the community to ensure the participation of both men and women. Finally, this study does not have a control group which did not participate in our walking class, which includes accelerometer measurements and learning soft-landing techniques. The absence of a control group prevents us from attributing the changes we observed in this study solely to the walking program. The interpretations should be limited to associations rather than causal conclusions. In the future we are planning to have a control group and compare the findings of this study with and without accelerometer measurements. Nevertheless, this study highlights the tools helping older adults learn soft-landing techniques in community walking classes.

## 5. Conclusions

In this study, landing impact during walking was successfully fed back to community walking participants as a relative load to body weight using upward acceleration. Measurements were smoothly conducted at multiple community centers, and participant comprehension and satisfaction were high. The effectiveness of the soft-landing technique taught in the walking lesson was confirmed using the accelerometer measurement data. In the future, longitudinal studies will be necessary to examine the sustainability of these effects and their relationship with knee or back pain.

## Figures and Tables

**Table 1:** 10 m gait assessment at pre- and post-walking lesson, *n* = 223.

Measurements	Pre-	Post-	*p*-Value	Z-Score	Effect Size (r)
Step length (body height ratio) *	0.32 (0.29–0.36)	0.31 (0.29–0.33)	<0.001	4.685	0.31
Step counts (per minute)	90.0 (84.0–96.0)	96.0 (87.0–102.0)	<0.001	6.036	0.40
Upward acceleration (m/s^2^) **	10.27 (9.43–11.50)	10.10 (9.48–10.75)	0.005	2.820	0.19
Walking speed (m/s)	1.37 (1.19–1.54)	1.35 (1.25–1.48)	0.899	0.127	0.01

* Step length (body height ratio): defined as the length of one step, measured from toe to toe, divided by the participant’s body height. ** Upward acceleration (m/s^2^): measured using an accelerometer placed at the lower back (Figure 4c). This value mainly reflects the impact strength that occurs during the load response phase while walking. It may indicate the magnitude of the impact strength transmitted to the lower back in the vertical direction.

**Table 2:** Multiple regression analyses for factors influencing upward acceleration.

Variable	Unstandardized Coefficients		Standardized Coefficients	*p*-Value	VIF
	B	SE	β		
Constant	−0.077	0.055		0.158	
Step length (body height ratio)	36.141	1.274	1.122	<0.001	7.018
Step counts (per minute)	0.082	0.007	0.286	<0.001	2.544
Walking speed (m/s)	1.842	0.459	0.168	<0.001	7.832

**Table 3:** Results of Survey Questions #6–#10 (*n* = 223).

Question	Yes (%)	No (%)	Not Answered (%)
Q#6. Did you know that the way you walk can increase or decrease the strain on your knees and lower back?	35.4	61.9	2.7
Q#7. Do you understand the meaning of the acceleration numbers?	96.4	0.0	3.6
Q#8. Do you want to improve your walking style by looking at the measurement results?	96.0	0.0	4.0
Q#9. Was the walking acceleration measurement useful?	96.0	0.9	3.1
Q#10. Would you like to take this measure again next time?	90.6	0.9	8.5

## Data Availability

The data presented in this study are available upon request.

## Appendix A

**Survey Questions**

**Question# 1.** While you are walking, which following items do you keep in mind? (Check all items that apply to you)

□ Walk with wider steps

□ Walk landing from your heels

□ Walk along a single straight line

□ Look ahead while you are walking

□ Walk with your back straight

□ Kicking the ground with your feet while you are walking

□ Walk fast

□ Avoid tripping while you are walking

□ Swing your arms while you are walking

□ Other: ________________________

**Question# 2.** Have you ever felt pain while walking?

□ Yes □ No

**Question# 3.** If yes, where did you feel pain? Check all items that apply to you. If it is not listed, write in Other.

□ Lower back □ Knees □ Hips □ Feet □ Other: ________________________

**Question# 4.** Have you ever had surgery on your knees, lower back, or other parts?

□ Yes □ No

**Question# 5.** If yes, where was the surgery site? Check all items that apply to you. If it is not listed, write in Other.

□ Lower back □ Knees □ Hips □ Feet □ Other: ________________________

**Question# 6.** Did you know that the way you walk can increase or decrease the strain on your knees and lower back?

□ Yes □ No

**Question# 7.** Do you understand the meaning of the acceleration numbers?

□ Yes □ No

**Question# 8.** Do you want to improve your walking style by looking at the measurement results?

□ Yes □ No

**Question# 9.** Was the walking acceleration measurement useful?

□ Yes □ No

**Question# 10.** Would you like to take this measure again next time?

□ Yes □ No

Free Comments: Please feel free to write any comments or requests regarding this walking program.

___________________________________________________________________________

___________________________________________________________________________

___________________________________________________________________________

## References

[1] Galloza J., Castillo B., Micheo W. (2017). Benefits of exercise in the older population. Phys. Med. Rehabil. Clin. N. Am..

[2] Landi F., Marzetti E., Martone A.M., Bernabei R., Onder G. (2014). Exercise as a remedy for sarcopenia. Curr. Opin. Clin. Nutr. Metab. Care.

[3] Gallaway P.J., Miyake H., Buchowski M.S., Shimada M., Yoshitake Y., Kim A.S., Hongu N. (2017). Physical activity: A viable way to reduce the risks of mild cognitive impairment, Alzheimer’s disease, and vascular dementia in older adults. Brain Sci..

[4] Thomas E., Battaglia G., Patti A., Brusa J., Leonardi V., Palma A., Bellafiore M. (2019). Physical activity programs for balance and fall prevention in elderly: A systematic review. Medicine.

[5] Sherrington C., Fairhall N., Kwok W., Wallbank G., Tiedemann A., Michaleff Z.A., Ng C.A.C.M., Bauman A. (2020). Evidence on physical activity and falls prevention for people aged 65+ years: Systematic review to inform the WHO guidelines. Int. J. Behav. Nutr. Phys. Act..

[6] Sherrington C., Fairhall N.J., Wallbank G.K., Tiedemann A., Michaleff Z.A., Howard K., Clemson L., Hopewell S., Lamb S.E. (2019). Exercise for preventing falls in older people living in the community. Cochrane Database Syst. Rev..

[7] Ploeg H.B., Bull F.C. (2020). Invest in physical activity to protect and promote health: The 2020 WHO guidelines. Int. J. Behav. Nutr. Phys. Act..

[8] Rössler R., Wagner J., Knaier R., Rommers N., Kressig R.W., Schmidt-Trucksäss A., Hinrichs T. (2024). Spatiotemporal gait characteristics across the adult lifespan. Gait Posture.

[9] Chung E., Lee S.-H., Lee H.-J., Kim Y.-H. (2023). Comparative study of young-old and old-old people using functional evaluation, gait characteristics, and cardiopulmonary metabolic energy consumption. BMC Geriatr..

[10] Tanaka R., Jung H., Yamashina S., Inoue Y.U., Nakamura R., Toda H., Imura T., Tamura H. (2022). Effects of age and gender on spatiotemporal and kinematic gait parameters in older adults. Acta Bioeng. Biomech..

[11] Ueno K., Kaneko H., Kamiya K., Okada A., Suzuki Y., Fujiu K., Matsuoka S., Michihata N., Takeda N., Jo T. (2023). Gait speed and cardiovascular disease by glycemic status. Am. J. Prev. Med..

[12] Windham B.G., Parker S.B., Zhu X., Gabriel K.P., Palta P., Sullivan K.J., Parker K.G., Knopman D.S., Gottesman R.F., Griswold M.E. (2022). Endurance and gait speed relationships with mild cognitive impairment and dementia. Alzheimer’s Dement..

[13] Woo J., Ho S.C., Yu A.L. (1999). Walking speed and stride length predict 36 months dependency, mortality, and institutionalization in Chinese aged 70 and older. J. Am. Geriatr. Soc..

[14] Ardestani M.M., Ferrigno C., Moazen M., Wimmer M.A. (2016). From normal to fast walking: Impact of cadence and stride length on lower extremity joint moments. Gait Posture.

[15] Edd S.N., Bennour S., Ulrich B., Jolles B.M., Favre J. (2020). Modifying stride length in Isolation and in combination with foot progression angle and step width can improve knee kinetics related to osteoarthritis; a preliminary study in healthy subjects. J. Biomech. Eng..

[16] Jang S., Lee K., Ju J.H. (2021). Recent updates of diagnosis, pathophysiology, and treatment on osteoarthritis of the knee. Int. J. Mol. Sci..

[17] Deshpande B.R., Katz J.N., Solomon D.H., Yelin E.H., Hunter D.J., Messier S.P., Suter L.G., Losina E. (2016). Number of persons with symptomatic knee osteoarthritis in the US: Impact of race and ethnicity, age, sex, and obesity. Arthritis Care Res..

[18] Yoshimura N., Muraki S., Oka H., Mabuchi A., En-Yo Y., Yoshida M., Saika A., Yoshida H., Suzuki T., Yamamoto S. (2009). Prevalence of knee osteoarthritis, lumber spondylosis, and osteoporosis in Japanese men and women: The research on osteoarthritis/osteoporosis against disability study. J. Bone Miner. Metab..

[19] Milner C.E., Meardon S.A., Hawkins J.L., Willson J.D. (2018). Walking velocity and step length adjustments affect knee joint contact forces in healthy weight and obese adults. J. Orthop. Res..

[20] Mao Q., Zheng W., Shi M., Yang F. (2024). Scientometric research and critical analysis of gait and balance in older adults: A scientometric review. Sensors.

[21] Ulrich B., Pereira L.C., Jolles B.M., Favre J. (2023). Walking with shorter stride length could improve knee kinetics of patients with medial knee osteoarthritis. J. Biomech..

[22] Gill N., O’Leary T., Roberts A., Liu A., Roerdink M., Greeves J., Jones R. (2023). Enforcing walking speed and step-length affects joint kinematics and kinetics in male and female healthy adults. Gait Posture.

[23] Lowry K.A., Vanswearingen J.M., Perera S., Studenski S.A., Brach J.S. (2013). Walking smoothness is associated with self-reported function after accounting for gait speed. J. Gerontol. Biol. Sci. Med. Sci..

[24] Ichikawa N., Doi T., Asada O. (1987). Building strength in the elderly. J. Clin. Sports Med..

[25] Doi T. (2010). Walking Safely to Extend Your Walking Life.

[26] Jung H., Yamashina S., Yamasaki R., Inoue Y., Hamada K., Hirohama K., Tanaka S., Tanaka R. (2023). Estimation of reference values of gait spatiotemporal and kinematic parameters in the lower extremities and trunk using markerless motion capture system for healthy older Japanese adults. Phys. Ther. Res..

[27] Simoneau G.G., Neumann D.A. (2010). Kinesiology of Walking. Kinesiology of the Musculoskeletal System.

[28] Wang Y., Mei Q., Jiang H., Hollander K., Van den Berghe P., Fernandez J., Gu Y. (2024). The biomechanical influence of step width on typical locomotor activities: A systematic review. Sports Med. Open.

[29] Akutagawa T., Enoki H., Takebayashi H., Murofushi Y., Oda S., Kondo H., Hosoda R., Nagano Y., Ikeuchi M. (2015). Validity of trunk accelerometric gait analysis: In comparison with analysis by force plate. J. Allied Health Sci..

[30] Osaka H., Watanabe S., Fujita D., Ishida H., Kobara K., Yoshimura Y., Ito T., Shinkoda K. (2011). Appropriate location of accelerometer for gait analysis: A comparative study based on cross-correlation coefficients. Rigakuryoho Kagaku.

[31] Takata K., Abo M. (2004). Gait evaluation with a small three-dimensional accelerometer. Tokyo Jikeikai Med. J..

[32] Rodríguez-Molinero A., Herrero-Larrea A., Miñarro A., Narvaiza L., Gálvez-Barrón C., Gonzalo León N., Valldosera E., de Mingo E., Macho O., Aivar D. (2019). The spatial parameters of gait and their association with falls, functional decline and death in older adults: A prospective study. Sci. Rep..

[33] Allet L., Ijzerman H., Meijer K., Wllems P., Savelberg H. (2011). The influence of stride-length on plantar foot-pressures and joint moments. Gait Posture.

